# Modelling the species jump: towards assessing the risk of human infection from novel avian influenzas

**DOI:** 10.1098/rsos.150173

**Published:** 2015-09-09

**Authors:** A. A. Hill, T. Dewé, R. Kosmider, S. Von Dobschuetz, O. Munoz, A. Hanna, A. Fusaro, M. De Nardi, W. Howard, K. Stevens, L. Kelly, A. Havelaar, K. Stärk

**Affiliations:** 1 Royal Veterinary College, London, UK; 2 Animal and Plant Health Agency, New Haw, Surrey, UK; 3 Food and Agriculture Organization of the United Nations, Rome, Italy; 4 Instituto Zooprofilattico Sperimentale delle Venizie, Padua, Italy; 5 RIVM, Bilthoven, The Netherlands

**Keywords:** avian influenza, risk assessment, zoonoses

## Abstract

The scientific understanding of the driving factors behind zoonotic and pandemic influenzas is hampered by complex interactions between viruses, animal hosts and humans. This complexity makes identifying influenza viruses of high zoonotic or pandemic risk, before they emerge from animal populations, extremely difficult and uncertain. As a first step towards assessing zoonotic risk of influenza, we demonstrate a risk assessment framework to assess the relative likelihood of influenza A viruses, circulating in animal populations, making the species jump into humans. The intention is that such a risk assessment framework could assist decision-makers to compare multiple influenza viruses for zoonotic potential and hence to develop appropriate strain-specific control measures. It also provides a first step towards showing proof of principle for an eventual pandemic risk model. We show that the spatial and temporal epidemiology is as important in assessing the risk of an influenza A species jump as understanding the innate molecular capability of the virus. We also demonstrate data deficiencies that need to be addressed in order to consistently combine both epidemiological and molecular virology data into a risk assessment framework.

## 1. Introduction

The interaction between animals and humans is a major source of emerging infectious diseases, some of which have the potential to cause human mortality, and some have and will cause global pandemics [1]. Zoonotic pathogens with pandemic potential include influenza A viruses, which caused the 1918 Spanish flu and 2009 H1N1 swine flu pandemics [2,3]. Indeed, the latter caught the scientific community by surprise as much attention had been focused on a pandemic originating from avian influenzas (AIs) such as highly pathogenic avian influenza (HPAI) H5N1. While there may still be potential for HPAI H5N1 to evolve into a pandemic virus, it is more accurately a waterfowl and poultry disease, which through widespread dissemination in poultry populations has managed to cause numerous human cases and fatalities. This example shows that we are still a long way from being able to accurately assess and distinguish between emerging, potentially zoonotic and potentially pandemic viruses. However, we must develop risk assessment tools to assess zoonotic and pandemic potential—at suitably early stages of emergence—if we are to efficiently allocate scarce resources on surveillance and controls.

Most pandemic pathogens originate in animals and are driven to emerge by ecological, behavioural or socio-economic changes [1]. Broadly speaking, pandemic potential can be realized through three distinct stages: first, the equilibrium of an animal disease is disturbed by ecological change (for example, human encroachment on wildlife habitat) that increases the likelihood of a species jump from the original animal host to another non-human wild species or livestock; second, local emergence in humans occurs through either sporadic cases or self-limiting person-to-person spread before dying out; finally, sustained person-to-person transmission occurs, enabling national or international spread of human disease and eventually a pandemic [1].

There is still much ongoing debate over the driving factors behind the emergence of influenza pandemics [3–5]; the science is hampered by complex environmental and evolutionary interactions and feedback loops. While contributory factors such as climate, farming practices and viral genetic reassortment have been postulated [3,5–7], their role in pandemic development remains unclear. Without a better appreciation of the epidemiology of pandemic development, any pandemic risk model will be subject to huge uncertainty. However, in order to work towards the goal of a tool that can be used to assess the risk of emerging influenzas causing zoonotic infections and eventually a pandemic, we can approach the task from first principles of disease transmission. We take the second stage of pandemic evolution, livestock–human infection, as a more manageable proof of principle (owing to better defined contact structures and relatively more data).

We therefore aim to address the problem of identifying which influenza strains are more likely to jump the species barrier and cause zoonotic infections, a prerequisite for a pandemic. Here, we describe the development of a risk assessment framework, based on general principles of disease transmission and risk, to assist decision-makers in their ability to rank influenza viruses, currently circulating in livestock, for their zoonotic potential. This will prioritize influenza strains potentially worthy of control measures. The earlier on in the chain of events a potentially zoonotic strain can be identified, the longer the warning and the more time available to prepare a vaccine or control the outbreak in animals. Our case studies relate to AI (where we have most data).

Influenza A has a natural reservoir in wild aquatic birds [2]. Both the HPAI H5N1 strain and the low-pathogenic avian influenza (LPAI) H7N9 strain that emerged in China in 2013 have wild bird origins [8,9] but are now found in domestic poultry. Human contact with infected poultry is believed to be the cause of the majority of human infections [10]. We have chosen HPAI H5N1 and China H7N9 as representative viruses that have reasonably good data available. We used associated molecular and epidemiological data to derive a model from mathematical first principles that can be applied to *any* influenza organism to assess the relative spatial risk of human infection. Essentially, we have built a prototype tool that has breadth of coverage, both spatially and across influenza strains, whereas previously most risk assessments and models have focused on detailed analysis of one single strain. We believe this makes our model much more amenable to decision-making and the allocation of resources. A novel viral molecular scoring system has also been incorporated into the model to characterize influenzas in their innate ability to cause human infection. We chose the case studies on the basis of available data and their relevance to sporadic, zoonotic infections, not pandemic potential (although both are still viewed with concern).

## 2. Methods

### 2.1 Model framework

We used standard disease transmission modelling principles [11] in order to assess the relative ability of individual virus strains circulating in animal populations to cause human infection. In essence, in order for zoonotic transmission to occur, there must be infected animals within effective transmission range of susceptible humans, where the number of contacts is proportional to the product of the number of infected animals and susceptible animals. There is some efficiency of transmission, which will vary according to the extent and intensity of contact between livestock and humans (e.g. commercial versus backyard production) and the innate efficiency of the virus to cause human infection (dependent on known or unknown genetic characteristics) [1,3,5]. These kinematics of infection imply two fundamental properties that must be considered when assessing risk: the opportunity for exposure of humans to the virus, and the capability of the virus to cause human infection. The former implies that any assessment of risk must be spatially dependent; the latter requires the development of a novel algorithm to assess the innate ability of a novel influenza virus to cause human infection, based on known genetic characteristics (see the electronic supplementary material and [12]).

The most appropriate model framework was to modify the classical epidemiological risk equation [13] which was then applied within a global spatial framework using standard geographic information system (GIS) methodology. First, we assessed the global contact intensity as a global indicator for the level of opportunity for exposure to humans from influenza viruses. The interactions between domestic and livestock animals and people will be different across the globe, but an intuitive contributing factor will be whether the animals are raised in a commercial or backyard production system. Therefore, an appropriate equation for the contact intensity within a cell *g*, *γ*(*g*), is

2.1 $$\gamma (g)=\sum_{j}w(\;j)C{\left(g,j\right)}^{2},$$

where we denote the number of animals of production type *j* (*j*={*commercial*,*backyard*}) within cell *g* by *C*( *g*,*j*) and the contact ratio with humans by *w*(*j*). It is important to note that this contact intensity is independent of influenza-specific parameters and hence is broadly applicable to all zoonotic organisms that depend on livestock–human interaction.

We assess risk by incorporating strain-specific information on the known location of livestock outbreaks along with an assessment of the innate capability of the virus to cause human infection. We therefore denote the risk of (one or more) human infections, given the presence of influenza strain *i* in cell *g* at time *t* as

2.2 $$R(i,g,t)∼\sum_{j=\{\mathrm{comm},\mathrm{back}\}}1-e^{-V(i)p(i,g,t)\tilde{\beta }(j)\gamma (g)}.$$

The transmission coefficient between chickens and humans is a summary term incorporating both host-specific ($\tilde{\beta }(j)$) and virus-specific (*V* (*i*)) components. The true prevalence of virus strain *i* at time *t*, *p*(*i*,*g*,*t*), was estimated by applying a spatial kernel to the number of outbreaks in a location within a six month period before and during the first human cases of a zoonotic outbreak, adjusting for under-reporting. That is, $p(i,g,t)∼\hat{n}(i,g,t)U(g)$, where $\hat{n}(i,g,t)$ is the normalized density estimator for cell *g*, and *U*(*g*) is the relevant under-reporting factor for HPAI or LPAI dependent on the type of surveillance conducted by a country (active or passive or both). The virus score, *V* (*i*), was determined using an algorithm based on the genetic factors most associated with the chronology of viral infection (attachment, replication, release).

The results of the model are intended to reflect the *relative* risk of human infection by comparing (novel) influenzas circulating in livestock populations: as such there must be a baseline from which to compare the relative risks. The normalization value is set as the maximum risk value achieved throughout the globe, which will be the cell with the highest contact intensity and *V* (*i*)=1. All other values are then normalized against this maximum risk value. Hence, the peak risk value of 2.57×10^−4^ for H5N1 clade 1 reported in the results is approximately 1/3890th of the maximum risk value possible.

### 2.2 Parameter estimation

The model framework was parametrized for domestic chicken to human transmission only; the initial intention was to include swine influenza as well, but data were lacking to be able to rigorously quantify parameters for this livestock species. We demonstrated proof of concept by estimating the zoonotic risk of HPAI H5N1 in southeast Asia in 2003–2004 and China H7N9 in 2013–2014 (i.e. at the time of their emergence in humans), although, in theory, the model is applicable to any influenza A strain and livestock species pairing.

**Figure 1.**
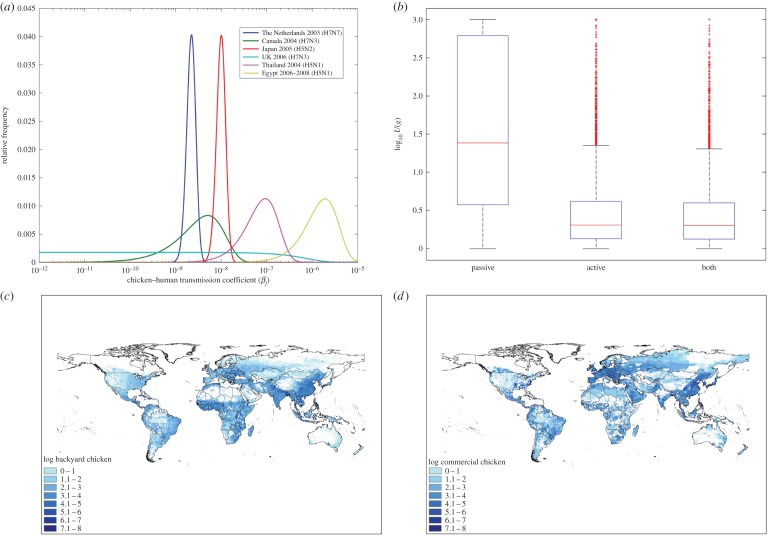
(*a*) Probability distributions for $\tilde{\beta }(\mathrm{back})$ (Egypt, Thailand) and $\tilde{\beta }(\mathrm{comm})$ (others), (*b*) under reporting factor distributions for active and combined surveillance systems (both HPAI and LPAI) and passive (HPAI only) (central red line, median; box, approx. 25th/75th percentiles; whiskers, approx. first and 99th percentile), (*c*,*d*) global domestic chicken population densities, backyard and commercial production systems, respectively [14].

Parameter estimates are given in table 1; key parameter estimates are also shown in figure 1. The genetic characteristics and the associated virus scores for two HPAI H5N1 strains (clades 1 and 2) from 2003 to 2004 and four China H7N9 profiles from 2013 are given in table 2.

**Table 1.** Summary of parameter estimates.

notation	description	value—mean (first, 99th percentile)
*C* (*comm*,*g*)	number of commercial domestic chickens per cell	6293 (0, 16883)
*C* (*back*,*g*)	number of backyard domestic chickens per cell	1654 (0, 6331)
*w* (*comm*)	contact ratio between commercial chickens and people	5.2×10^−4^ (4.8×10^−5^, 9.8×10^−4^)
*w* (*back*)	contact ratio between backyard chickens and people	0.51 (0.03, 0.99)
$\tilde{\beta }\;(\mathrm{comm})$	transmission parameter: commercial chickens → humans	2.03×10^−7^ (1.16×10^−9^, 1.32×10^−6^)
$\tilde{\beta }\;(\mathrm{back})$	transmission parameter: backyard chickens → humans	1.63×10^−6^ (6.05×10^−8^, 5.62×10^−6^)
$\hat{n}\;(i,g,t)$	normalized density estimator	see electronic supplementary material
*U* (*active*)	under-reporting factor for active surveillance of HPAI and LPAI	8.2 (1.0, 99.7)
*U* (*passive*)	under-reporting factor for passive surveillance of HPAI	286.1 (1.0, 1000.0)
*U* (*both*)	under-reporting factor for both active and passive surveillance of HPAI and LPAI	7.7 (1.0, 108.4)

**Table 2.** Summary of virus score characteristics for H5N1 clade categories 1 and 2, and four H7N9 profiles.

strain	virus score, *V* (*i*) (5th; 95th percentile)	receptor preference	presence of known mutations	reassortments within virus	stalk deletion in NA	phylogenetic relatedness of HA to HA of strains circulating in humans
H5N1 clade 1	0.27 (0.21; 0.33)	*α*2,3	mutations	none	long stalk	different
H5N1 clade 2	0.08 (0.03; 0.14)	*α*2,3	none	none	long stalk	different
H7N9 profile 7	0.60 (0.58; 0.61)	*α*2,6	none	acquisition	short stalk	different
H7N9 profile 15	0.84 (0.78; 0.89)	*α*2,6	mutations	none	short stalk	different
H7N9 profile 23	0.30 (0.23; 0.36)	*α*2,3	none	acquisition	short stalk	different
H7N9 profile 31	0.53 (0.43; 0.64)	*α*2,3	mutations	acquisition	short stalk	different

Commercial and backyard chicken population density at a resolution of 0.08 pixels (between 5 and 80 km^2^ depending on latitude) was supplied by the Food and Agriculture Organization of the United Nations (FAO; figure 1). A full description of the density estimates is given in the accompanying paper to the ongoing FAO Gridded Livestock of the World project [14]. Briefly, reported subnational livestock statistics are collected and cleaned. Appropriate habitats for domestic chickens (commercial and backyard) were identified by applying various GIS masks (e.g. excluding lakes and steep mountains as suitable habitats). Livestock densities are then calculated by implementing spatially stratified, statistical regression models that test a number of predictor environmental variables for relevance. The main spatial dataset used in the statistical modelling is a Fourier-processed decadal time series of geophysical variables derived from moderate resolution imaging spectroradiometer satellite data from 2001 to 2008. The variables include two vegetation indices, land surface temperature and the band 3 middle-infrared, which is used to assist in vegetation mapping. Predicted densities are then compared and adjusted against livestock statistics to provide a final validation. All spatially dependent parameters used in the model were resampled to match the commercially reared density dataset (at an approximate resolution of 0.08°). Population densities were then split into commercial and backyard chicken production types by use of gross domestic product per capita statistics [15].

The relative weighting of contact between chickens and humans (*w*_*j*_) will be different for commercial and backyard chickens, where the former will be reared by relatively few people compared with backyard chickens, which will be reared by hand by one or two people. We assume that the ratio of chicken to humans for backyard chickens is 1 : 1 [16] and of the order of 10 000 : 1 for commercial production (assuming that commercial flock sizes are in the tens of thousands, and there would be a small number of farm workers looking after each flock). We have placed some uncertainty around these average contact ratios based on our own intuition (between 1 : 1 and 50 : 1 for backyard production and between 1000 : 1 and 25 000 : 1 for commercial production). For the contact intensity model, where values are normalized and hence the variation in contact ratios has no effect on the relative values, point values of 1×10^−4^ and 1 were used for commercial and backyard contact ratios, respectively.

The epidemiological component of the transmission parameter ($\tilde{\beta }(j)$) was generated from published real-world reports of AI outbreaks [17–27]. The normalized virus density, ${\hat{n}}_{i}$, was estimated by applying a spatial kernel estimator to extracted livestock species and location data of AI outbreaks from the Empres-i animal disease information database maintained by FAO [28], which was merged with information of genetic sequencing from OpenFluDB, a publicly available influenza-specialized database developed by the Swiss Institute for Bioinformatics that contains genomic and protein influenza virus sequences [29]. The under-reporting factors for active and passive surveillance of HPAI and LPAI were estimated from several studies that have estimated the sensitivity of active and passive surveillance systems (SSe) for individual countries. For example, the overall distribution for HPAI passive surveillance was generated from resampling from individual distributions for Spain, New Zealand, Nigeria and Denmark [30–32]. Further information on the derivation of parameter estimates, including the transmission parameter, virus score and under-reporting factor, is given in the electronic supplementary material and [12].

### 2.3 Uncertainty analysis

There are a number of uncertain parameter estimates within the model ($\tilde{\beta }(j)$, *w*(*commercial*) and *V* (*i*)). For the uncertainty analysis the 25th, 50th, 75th and 99th percentile values of the product of 10 000 random samples from each of these distributions are used as inputs into the rest of the risk equation (equation (2.2)). The end result is a highly uncertain output for *R*(*i*,*j*,*t*), stretching over several orders of magnitude from the 25th to 99th percentile. Most of the uncertainty range is attributable to the transmission parameter $\tilde{\beta }(\;j)$, which has a range over six ( *j*, commercial) and three ( *j*, backyard) orders of magnitude (figure 1).

## 3. Results

### 3.1 Global contact intensity, *γ*(*g*)

**Figure 2.**
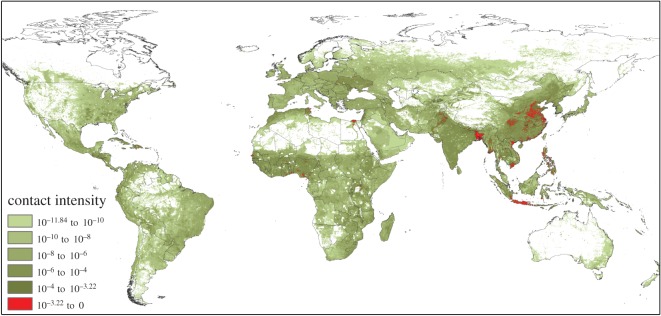
Normalized contact intensity map for domestic chicken–human interaction across the globe. Red areas indicate the top 10% of cells with regards to contact intensity, which represents approximately 92% of all global contacts.

**Figure 3.**
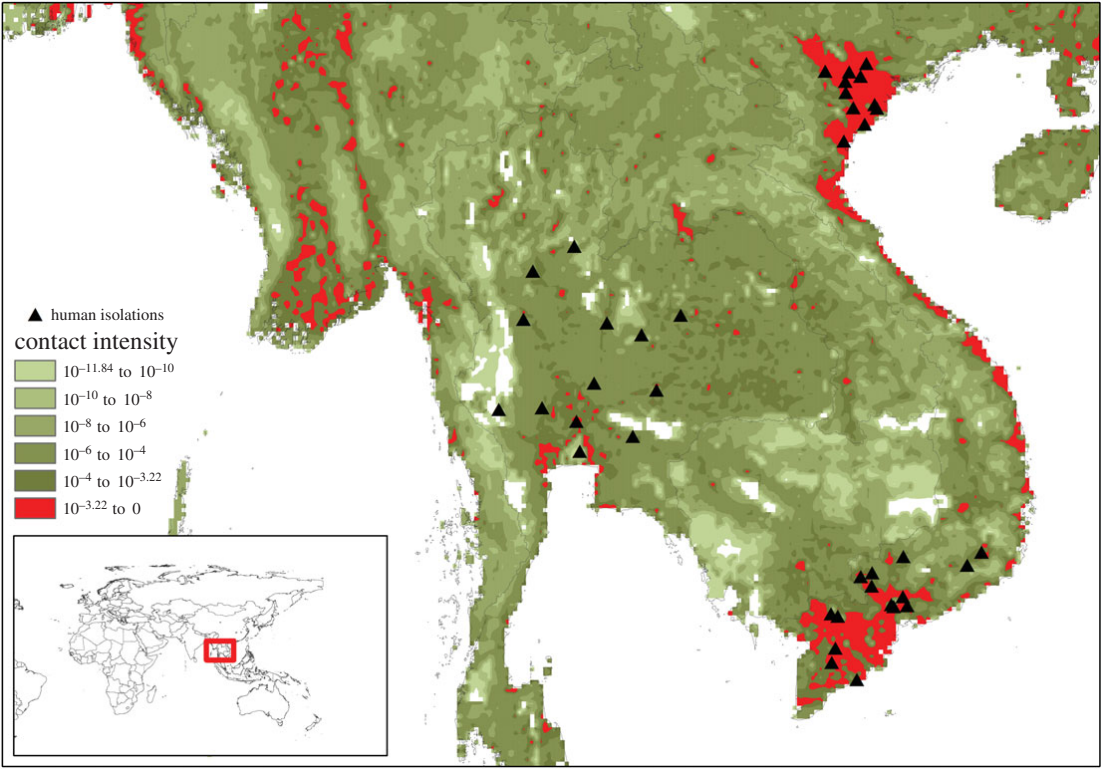
Exploded view of contact intensity map in southeast Asia region, overlaid with human isolations of HPAI H5N1 in the 2003–2004 outbreak. Only records with longitude/latitude coordinates are included.

By mapping contact intensity, we have identified the high-risk areas for *any* zoonotic infection that relies on human interaction with domestic chickens (figure 2). This simple method, converting chicken population density into contact intensity with humans, can be used to target surveillance for zoonotic diseases in chickens and is also applicable to other livestock species. While high-opportunity areas for AI spillover in southeast Asia and the Nile delta are well known, the quantification of contact intensity highlights the considerable global heterogeneity of zoonotic risk: cells with contact intensity greater than the 99th percentile contribute approximately 46% of all global contacts between domestic chickens and people, whereas cells greater than the 90th percentile contribute approximately 92% of total global contacts. Almost 90% of species jump opportunities reside within an area the same size as Switzerland. As a clear example of the importance of contact alone (regardless of virus fitness to infect humans), cases of HPAI H5N1 during the 2003–2004 outbreak in southeast Asia map remarkably closely to some of the highest contact intensities in the region and the globe (figure 3).

### 3.2 Relative risk map

The second output from the model, the relative risk map, is critical to identifying emerging influenza threats before spillover into humans occurs. It uniquely and quantitatively distinguishes between the risks presented by individual virus strains. For the HPAI H5N1 outbreak in 2003–2004, it was possible to characterize the risk of two virus strains (H5N1 clades 1 and 2) in southeast Asia. Human cases were highly spatially clustered (figure 3). Both H5N1 clades were given relatively low virus scores (table 2 and electronic supplementary material), which indicates a low inherent ability to infect people. Clade 1 viruses scored slightly higher than clade 2 owing to the presence in a clade 1 isolate of potentially significant mutations. Despite these low virus scores, the high rate of interaction between domestic chickens and people in southeast Asia provided sufficient opportunity for significant bird-to-human infections. The results of our model agree well with the interpretation of a recent prospective study of HPAI H5N1, which suggests that the zoonotic risk of this subtype is much like any other AI strain; the difference being that HPAI H5N1 is widely spread around the globe and hence has much more contact with humans [33,34].

**Figure 4.**
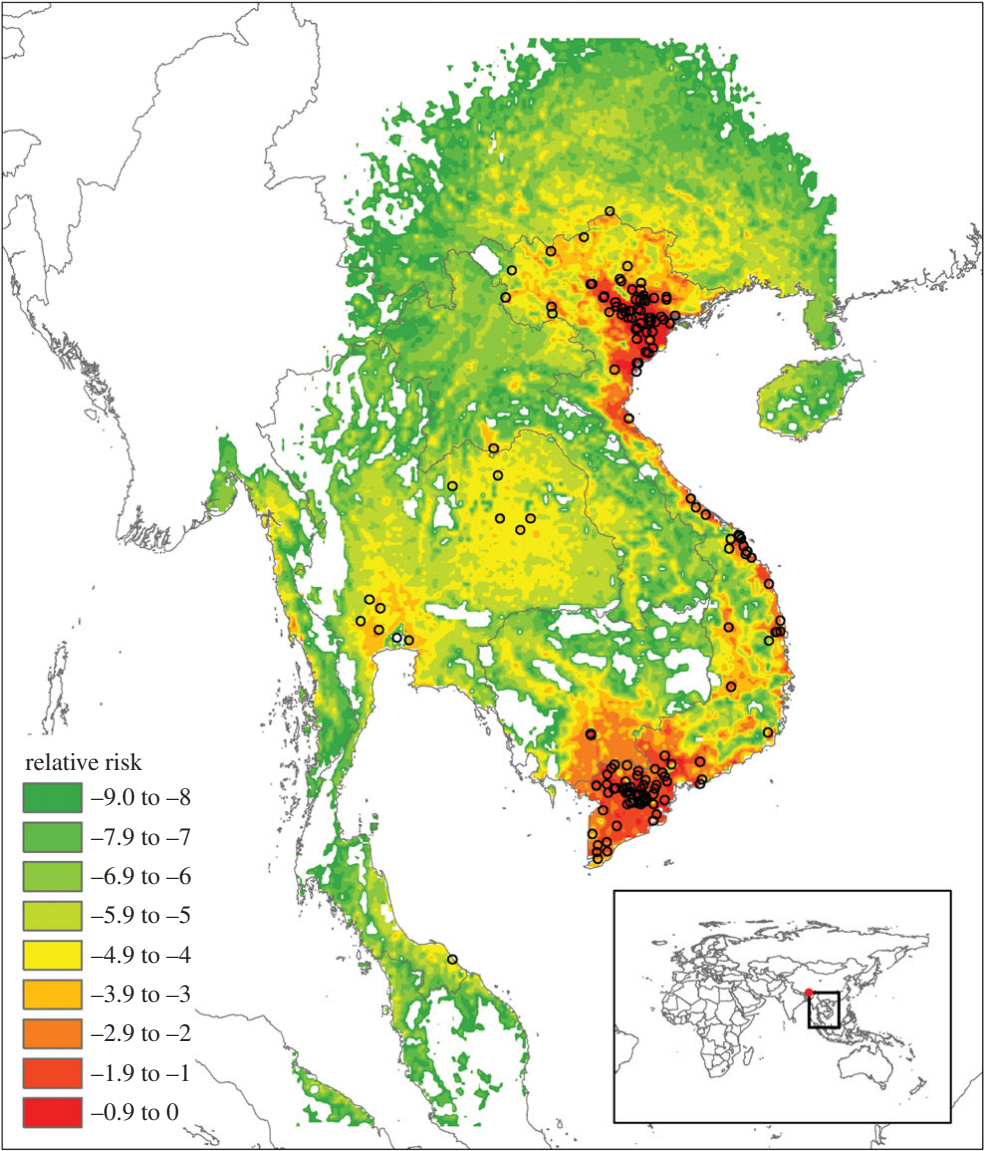
Example of relative risk map shows the relative spatial likelihood of one or more human infections for HPAI H5N1 clade 1, for the six months prior to 20 May 2004 (99th percentile risk values shown for clarity). Relative risk on log_10_ scale. Black circles represent outbreaks with known longitude and latitude coordinates.

**Figure 5.**
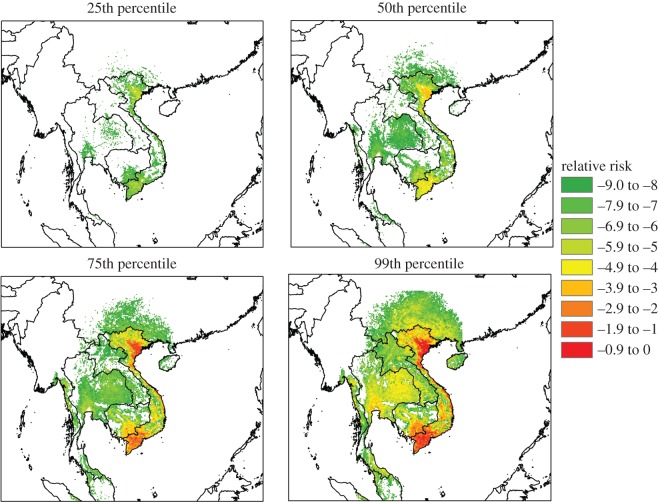
Uncertainty in zoonotic risk of H5N1 clade 1 represented by postage stamp maps. The majority of the variation in relative risk is due to the epidemiological transmission parameter.

The overall risk map for clades 1 is shown in figure 4. A spatial kernel was used to estimate the density of H5N1 clades 1 and 2 based on the location of outbreaks with known longitude/latitude coordinates. There was large uncertainty in the results of the risk map (figure 5), but the most important output of the framework, the relative ranking of viruses, should be reasonably well preserved, given that the majority of uncertainty derived from the ‘generic’ virus transmission parameter.

Summary statistics for clades 1 and 2 are that the median peak normalized risks per cell for clades 1 and 2 were 1.12×10^−3^ and 1.10×10^−4^, respectively; average risks per cell at the 50th percentile for clades 1 and 2 were 2.95×10^−7^ and 5.01×10^−8^, respectively. The important result of this normalized risk ranking is the relative difference between the two clades. The impact of the spatial context of the two clades' distributions is apparent: the threefold difference in virus score between clade 1 and 2 (table 2) is magnified by the spatial differences, resulting in average and peak risk values that are six and 10 times higher, respectively, for clades 1 than 2. We should therefore expect that H5N1 clade 1 is *more likely* to jump into humans than clade 2. Within the FAO Empres-i database [28], 288 human isolates were recorded during 2003–2004 that we were also able to match to clade information available from the human and animal influenza virus OpenFlu database [35]. Of these, 159 were classified as clade 1 and 74 as clade 2, thus suggesting than clade 1 isolates were more likely to cause human infection.

The second case study was the current China H7N9 outbreak. Most H7N9 isolates of animal or environmental origin were isolated as a result of targeted surveillance of poultry markets *after* human infections were identified. Hence, there is such a large bias towards the location of animal and environmental isolations in the apparent spatial distribution for H7N9 that it is not prudent to run the full model for this low-pathogenic virus. We can, however, assume that the spatial distribution in chickens was not negligible before human infection. The low pathogenicity of H7N9 demonstrates the challenges in identifying zoonotic influenzas before the occurrence of human cases, and the need to conduct active, risk-based surveillance for low-pathogenic strains [36].

It was nevertheless a worthwhile exercise to calculate the virus score for the H7N9 viruses isolated from animals and poultry markets (see [37] for reference to many of the animal and environmental isolates used in our analysis). H7N9 is a well-characterized amalgamation of H7 and N9 surface proteins from wild birds, with internal proteins reassorted from H9N2 poultry viruses [9,38,39]. As previous studies have also shown, we identified four different virus profiles for the H7N9 isolates (see electronic supplementary material). All of these H7N9 profiles scored much higher than the H5N1 clades because of their enhanced ability to bind to cells in the human upper respiratory tract, and the presence of potentially significant mutations enabling more efficient human infection (table 2).

## 4. Discussion

The inclusion of quantitative data regarding animal production, surveillance systems and geographical location of viruses makes this prototype model the first truly global and risk-based assessment of the zoonotic potential of influenza A viruses, and addresses many of the requirements thought necessary to assess the transition from pre-emergence to local emergence, as recently defined by Morse *et al.* [1]. The model developed here captures many of the crucial differences in the dynamics of zoonotic transmission of HPAI H5N1 and LPAI H7N9. HPAI H5N1 is now considered endemic in poultry in several countries (e.g. Egypt, China, Indonesia), but this has translated into a lower global incidence of human cases than H7N9, which has only been isolated (so far) from a limited geographical area. This is consistent with an H5N1 virus that is much less efficient in causing human infection than H7N9. We highlight that the two case study viruses, each at one time feared to be a potential precursor to a pandemic virus, are characterized as essentially still *avian* influenzas, which have simply found new niches to exploit. Other factors beyond the number of sporadic human cases are likely to be more predictive of potential pandemic potential. However, the results indicate that our method could be used to provide an initial but robust tool for projecting which currently circulating animal influenzas pose a greater zoonotic risk, and where.

The roles of wildlife and poultry markets, both indicated as risk factors for transmission to chickens or humans respectively [40,41] are not explicitly included within the current model. Rather, the presence of poultry markets is captured implicitly, as (i) higher human population density is closely linked to the presence of poultry markets in southeast Asia [42,43] and (ii) chicken population density is positively correlated with human population density [16]. Wild birds have been shown to be directly responsible for a few human cases of HPAI H5N1, but the majority of both H5N1 and H7N9 cases in humans are linked to contact with domestic poultry [10]. We therefore believe that this implicit approach is valid for the broad framework developed here, which is designed to be a reasonably simple model that can be deployed rapidly and globally.

The quantification of the inherent ability of different virus strains and subtypes to cause human infection is a challenge, as molecular determinants of transmissibility, stability and host range are still not fully understood. However, while further knowledge of the molecular biology of a virus will hopefully in time help to distinguish viruses that are more or less likely to establish productive human infections, knowledge of the epidemiological situation (e.g. the prevalence of a virus in animal populations and the type of interactions between human and animals) is still vital in determining the risk of a future species jump (or indeed pandemic). Therefore, we must align molecular virology data with livestock, physical and environmental metadata to produce rigorous risk-based frameworks.

The combination of molecular data with spatial and geographical information has the potential to yield unique insights into the evolution and epidemiology of pathogens such as influenza. Spatial, meteorological and/or population data can be used to define ecological or zoonotic niches, for example assessing the likely areas for zoonotic West Nile or Ebola virus transmission before or during introduction of the virus [44–46]. The inclusion of molecular data, such as in this model, allows us to differentiate at a much higher resolution than previously, while also allowing for potentially more insights by, for example, associating phenotype or genotype with environmental factors, or tracing the environmental drivers behind the emergence of a particular strain. However, such unique knowledge can be gained only by the rigorous (public) collation and standardization of data from outbreaks, surveillance and research, which is currently lacking, despite the recent development of international databases such as GenBank [47].

Current surveillance activities for influenzas in animals are focused on facilitating trade, and there are only a few programmes intended specifically to monitor potentially zoonotic influenzas, with these few only for intensive swine production [36]. However, these efforts to identify pre-pandemic viruses are also stymied by current non-standardized data collection practices during outbreaks or routine surveillance [36]. Indeed, much of our efforts in assessing zoonotic risk during the development of this model were placed in realigning the metadata (e.g. species affected, geographical location of outbreak) with the genetic sequencing information we derived from OpenFlu and GenBank for H5N1 and H7N9 isolates. Despite improvements in centralizing data collection efforts since the HPAI H5N1 outbreak in 2004 onwards, we were only able to fully characterize 33% of relevant LPAI H7N9 isolates. This lack of complete data forms a major impediment to progress in understanding the interactions between the environmental and molecular factors that drive influenza transmission, as both characteristics of a virus isolate are required to understand the epidemiology of a novel virus strain.

Large uncertainties in the data used for the current model were identified, especially in the efficiency of virus transmission from chicken to human. We therefore recommend standardization of genetic and metadata collation during outbreaks as a first priority—much more could be done to use data *already being collected* to investigate the epidemiology of zoonotic influenzas if the infrastructure existed to capture data in as complete and centralized form as possible. Further specific surveillance for zoonotic influenzas would of course be helpful (and the current model suggests areas of the globe in which to focus domestic chicken surveillance), but in the short term a better understanding of effective (i.e. transmissive) interactions between chickens and people might be more achievable, and would provide more reliable estimates of the model's transmission parameters.

Previous risk assessments of zoonotic influenzas have tended to focus on identifying the molecular characteristics of the strain, the presenting signs and/or the epidemiological risk factors *after* human infections have occurred [48–50]. Most are qualitative and reactive, although there are more sophisticated spatial analyses, for example trying to predict the likely locations of further human H7N9 cases based on the occurrence of poultry markets [51]. While statistical correlation is a useful method for describing spatial risk in the context of a *specific* organism, it does not readily assist (without large simplifying assumptions) when trying to identify and rank the risks of multiple strains, as decision-makers will have to do in order to prioritize which strains are monitored and/or controlled. The same issue applies to several theoretical models that have been developed describing the (spatial) spread of AI in poultry and humans [52–55]; these models provide useful ways to assess (i) how spread may occur in different circumstances and (ii) how spread may be mitigated under different control strategies, but are reasonably complex and focus on one generic strain of influenza. In addition, many of these models remain largely theoretical owing to a lack of precise parameter estimation for a number of reasonably obscure or hard-to-measure parameters. We face similar problems of parameter estimation; however, our focus on a relatively simple model that requires reasonably accessible parameters does somewhat mitigate this common problem. This allows us to be reasonably confident in the results of the epidemiological component of the model, although we still face difficulties with parameter estimation of the molecular component of the model owing to a lack of knowledge about the genetic mechanisms that drive human infection. However, the explicit consideration of different strains does allow our model to better realize the aim of being used as a decision support tool that has breadth of coverage across variation in strain and space.

## 5. Conclusion

Anticipating the spillover of influenza viruses from animals into people is a daunting task; anticipating the development of a pandemic influenza is more daunting still. Our efforts serve to mark the first truly quantitative attempt to characterize the global risk of a species jump into humans, one of the defining moments of the pathway from an innocuous pathogen of wildlife to pandemic virus. Even taking this broad, reasonably simple model of the human spillover stage (arguably the best understood of the three stages as outlined by Morse *et al*. [1]) demands almost all readily available data on chicken populations and AI surveillance. It is therefore clear we are some way from being able to assess pandemic risk in a similar fashion, as even more known and unknown factors would need to be considered and parametrized. However, the exercise of developing and using such a model assists the progression of scientific knowledge by identifying data gaps and inconsistencies in data collection practices, as well as challenging perceived wisdoms.

To be able to successfully apply our model to zoonoses prevention and control, the epidemiological and molecular inputs need to be immediately accessible. In addition, a risk assessment framework should be transferrable between viral strains, livestock species and geographical areas, and flexible enough to respond to new information. The generic risk assessment framework we have developed, focusing on local emergence of livestock–human transmission, simplifies and systematizes the current extent of our knowledge around such spillover events, and remains applicable to many livestock/virus species combinations. It also provides immediate information to disease control professionals on the global risk of influenza transmission. The model has been tested using domestic chicken and two well-known AIs, and will be further trialled within FAO's own risk assessment systems.

## Supplementary Material

Supplementary information PDF - contains extra information allowing reader to recreate parameterisation of model.

## Supplementary Material

FLURISK H5N1 virus analysis.xls - characterisation of matched H5N1 isolates

## Supplementary Material

H7N9 linkages.xls - example of matching process between Empres-i metadata and genetic sequencing reference numbers.

## Supplementary Material

Final country surveillance.csv - Provides the AI surveillance type for each country as used in the model.

## Supplementary Material

Results.zip

## Data accessibility

The model was developed in R v. 3.0 (R: a language and environment for statistical computing; R Foundation for Statistical Computing, software available for download at http://www.R-project.org/). Additional packages used included Logit, BigMemory, Sp, RGDAL and spatial. The IP of the R code is currently held by EFSA; the code is available for use upon agreement by EFSA and the FLURISK project team partners. The population density data for commercial and backyard chickens was produced by the FAO, using preliminary datasets. GIS datasets of the final global population densities will be available on the Livestock GeoWiki as they are completed (http://www.livestock.geo-wiki.org). The country surveillance types are listed in ‘Country surveillance types.csv’. The records used to define the spatial distributions of HPAI H5N1 clades 1 and 2 are freely available and were downloaded from FAO's Empres-i website, at http://empres-i.fao.org [56]. We were able to match isolates from the Empres-i database to genetic sequencing information in GenBank for a limited number of H5N1 clade 1 and 2 isolates; these are listed, along with the characterization of the virus scores, in the supplementary file ‘FLURISK H5N1 virus analysis.xls’. A similar process was conducted for the H7N9 isolates; see ‘H7N9 linkages.xlsx’. It can be seen from these datasets that incomplete or unvalidated data collection means we are unable to match all isolates. The GIS .tif files associated with figures 4 and 5 are provided in the ‘Results.zip’, which also includes a .tif file of the spatial risk result for H5N1 clade 2 (50th percentile result). We also provide the first, 25th, 75th and 99th percentile results for H5N1 clade 1 as an example of the uncertainty generated through the analysis.
