# Supplementary material for: Modelling the species jump: towards assessing the risk of human infection from novel avian influenzas
Source: R Soc Open Sci. 2015 Sep 9;2(9):150173. doi: 10.1098/rsos.150173 (PMC4593676; doi:10.1098/rsos.150173)
Supplement: Supplementary information PDF - contains extra information allowing reader to recreate parameterisation of model. [file rsos150173supp1.pdf]

Supplementary information

Detailed parameter estimation

A detailed description of the methodologies used to derive all parameter estimates is given in the external FLURISK project report to the European Food Safety Authority (EFSA) [17]. Here we briefly describe some of the most important parameters and the estimation used in the paper (in some cases different to the original report).

Parameter estimates are listed in Table 1 of the main text. Key parameter estimates are also shown in Figure 1.

Global domestic chicken population density

Commercial and backyard chicken population density at a resolution of 0.08 pixels (between 5-80km^2 depending on latitude) was supplied by the Food and Agriculture Organization of the United Nations (FAO) [19] (see Figure 1 in main text). All spatially-dependent parameters used in the model were resampled to match the commercially-reared density dataset (at an approximate resolution of 0.08 degrees). For the contact intensity model, where values are normalised and hence the variation in contact ratios has no effect on the relative values, point values of 1×10^{-4} and 1 were used for commercial and backyard contact ratios respectively.

Epidemiological transmission parameter, $\tilde{\beta}(j)$

The epidemiological component of the transmission parameter ($\tilde{\beta}(j)$) was generated from published real-world reports of Avian Influenza (AI) outbreaks (see Table 1) in chickens that resulted in human infections. Introducing an appropriate form of distribution to capture uncertainty,

$$\tilde{\beta}(j) \sim Gamma\left(I_H, \frac{1}{S_H I(j)}\right), \tag{1}$$

where I_H and S_H are the number of infected and susceptible humans and $I(j)$ is the number of infected chickens of type j in an outbreak.

The information necessary to parameterise the transmission parameter was available for a few high-profile AI outbreaks in commercial production systems. Only humans that were exposed during routine

Table 1. Parameter estimates for the epidemiological component of the transmission parameter (see Equation (1)), taken from four avian influenza outbreaks in intensively reared production systems and two outbreaks in extensively reared production systems.

Outbreak	Production type	I_j	I_H	S_H	References
Netherlands 2003 (H7N7)	Commercial	6,096,898	20	1,400	[20,21]
Canada 2004 (H7N3)	Commercial	1,147,108	2	168	[22,23]
Japan 2005 (H5N2)	Commercial	5,700,000	20	332	[24]
United Kingdom 2006 (H7N3)	Commercial	47,500	1	26	[25]
Thailand 2004 (H5N1)	Backyard	Bin($6.3*10^7$,$4.8*10^{-4}$)	14	Poisson(1019×4)	[26–28]
Egypt 2006-2008 (H5N1)	Backyard	Poisson(606×15.2)	57	Poisson(606×4.6)	[29,30]

contact with birds (e.g. farmers and farm workers) were included, and not those taking part in post-influenza-detection interventions (e.g. government workers engaged in systematic culling). The number of infected birds is not usually specified in epidemiological reports of such outbreaks, so the total number of birds on infected farms was used as a proxy. The number of infected birds and exposed people were not available for outbreaks involving backyard flocks, so these were simulated by selecting the best fit to crude estimations of household size and poultry holdings (for example, for the Egypt outbreak the number of infected chickens in the outbreak was estimated by the number of households involved (606) and the average number of chickens per household (15.2) [30]).

The contact ratios for chicken: human in commercial and backyard production were generated from $w\,(comm) \sim \text{Uniform}\left(4 \times 10^{-5}, 1 \times 10^{-3}\right)$ and $w\,(back) \sim \text{Uniform}(0.02, 1)$ respectively.

Normalised virus density, $\hat{n}_i$

In order to estimate the normalised virus density, $\hat{n}_i$, we extracted livestock species and location data of AI outbreaks from the Empres-i animal disease information database maintained by FAO (`http://empres-i.fao.org/eipws3g/`), and then merged it with information of genetic sequencing from OpenFluDB, a publicly available influenza-specialized database developed by the Swiss Institute for Bioinformatics (SIB) that contains genomic and protein influenza virus sequences (`http://openflu.vital-it.ch/browse.php`).

For HPAI H5N1, six months' data were extracted from the Empres-i database, including all infected farms recorded during the poultry and human outbreak in South-East Asia during the first human isolations of H5N1 throughout 2003-2004 (records taken from 1st December 2003 to 31st May 2004). There are over 4000 records for H5N1 from Empres-i during this six-month period, and another 400+ from OpenFluDB. Unfortunately neither database records information with regard to the virus sequence identified from any of the outbreaks (or references Genbank accession numbers), although OpenFluDB does record the specific clade of H5N1 isolated. Hence, for the purposes of this assessment, we have categorised the H5N1 isolates by clade (grouping by primary number, to ensure that enough isolates are included to provide a reasonable assessment of spatial distribution). While the Empres-i database includes standardised field names and data requirements, many records are incomplete or have limited information regarding geographical location and/or species affected. A pilot study undertaken at FAO has merged the two Empres-i and OpenFluDB datasets together, providing 422 outbreaks that could be matched under both databases, and which included information on the specific clade of H5N1. A subset of the first 25 records from the dataset is given in Table 2; the full dataset is available as supplementary file H5N1_clade_epidata.csv.

It is clear from Table 2 that there are several issues with the data standardisation and format. A subset of this dataset was taken and modified in order to run through the model. First, only domestic chicken outbreaks were included (i.e. Fields "Species 1"=domestic and "Species 2" included "chicken"). Second, clades were grouped as described in the main text, into clade categories. Only clade categories with more than 20 recorded outbreaks were used in order to ensure a reasonable distribution of spatial points (leaving Clades 1, 2, 5 and 9). For the purposes of clarity in presentation, we focused on the most numerous clades only, Clades 1 and 2. For both clade categories 1 and 2 a bounding box was set up using the furthest available north-westerly and south-easterly co-ordinates (plus or minus five degrees latitude/longitude as a reasonable extension to capture any unrecorded outbreaks); records with no longitude or latitude information were then randomly assigned co-ordinates within the bounding box prescribed by the outermost co-ordinates to increase the number of records available to input into the model. A spatial kernel density model was applied to the spatial point data for each H5N1 clade to weight and normalise the spatial intensity of infection (we used the function bkde2D from R package 'KernSmooth', with a bandwith of 1 in each direction). The resulting spatial distribution of virus density for each clade grouping is shown in Figure 1.

Table 2. Subset of data from linked EMPRES-i and OpenFlu databases for HPAI H5N1 outbreaks in South-East Asia in 2003-2004 (first 25 records with clade information recorded).

ID	Lat	Long	Country	Admin 1	Observation date	Clade	Species 1	Species 2
1			China	Fujian	2004-00-00	6	Wild	Bird
2			China		2004-00-00	7	Human	
3			Thailand		2004-00-00	1	Unspecified	Bird
4			China		2003-00-00	1	Wild	Gull
5			China	Hunan	2004-00-00	6	Unspecified	bird
6			Japan	Kyoto-fu	2004-00-00	2.5	Insect	Insect
7			Thailand		2004-00-00	1	Mammal	Cat
8*	13.923	100.108	Thailand		08/11/2012	1	Domestic	Chicken
9			Thailand		2004-00-00	1	Domestic	Chicken
10			China	Anhui	2004-00-00	9	Domestic	Chicken
11			Indonesia		2004-00-00	2.1.1	Domestic	Chicken
12			Indonesia		2004-00-00	2.1.1	Domestic	Chicken
13			Indonesia	Propinsi	2004-00-00	2.1-like	Domestic	Chicken
14*	11.56	104.89	Cambodia	Phnom Pet	12/01/2004	1	Domestic	Chicken
15			China		2004-00-00	2.4	Domestic	Chicken
16			Indonesia	Propinsi	2004-00-00	2.1.1	Domestic	Chicken
17			Indonesia	East Java	2003-10-00	2.1.1	Domestic	Chicken
18			Indonesia	East Java	2003-11-15	2.1.1	Domestic	Chicken
19			Indonesia	East Kalio	2004-02-16	2.1.1	Domestic	Chicken
20			China	Gansu Sh	2004-00-00	9	Domestic	Chicken
21	36.227	103.801	China	Gansu Sh	30/01/2004	9	Domestic	Chicken
22			China	Guangdom	2004-00-00	2.5	Domestic	Chicken
23			China	Guangdom	2004-00-00	2.3.2	Domestic	Chicken
24			China	Guangdom	2004-00-00	2.3.1	Domestic	Chicken
25	25.118	114.312	China	Guangdom	06/02/2004	2.3.1	Domestic	Bird

Estimation of under-reporting factor

The full derivation of Equation (3) is given in [17]. The number of AI-infected farms detected in a country using a surveillance system with sensitivity SSe, N_D is given by

$$N_D = N_S \left(1 - (1 - SSe)^{-N_S}\right), \tag{2}$$

where N_S is the number of farms sampled in an active surveillance system or the number of farms in the country if practicing only a passive surveillance system. The under-reporting factor for the virus density in a cell, U, is then given by

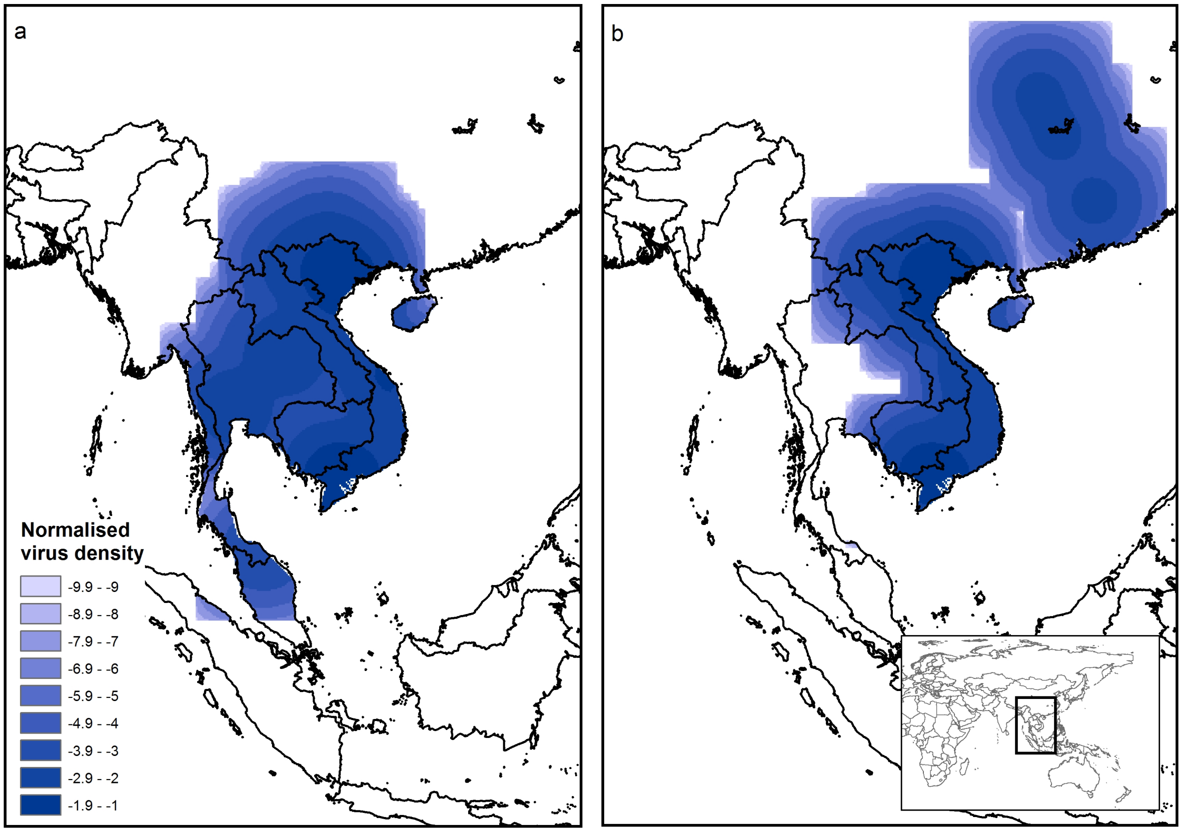

Figure 1. Normalised virus density (log10 scale) for a) HPAI H5N1 Clade 1 and b) HPAI H5N1 Clade 2 in South-East Asia between December 2003 - May 2004.

$$U \sim \frac{1}{Beta\left(N_D, N_S - N_D\right)}. \tag{3}$$

Various studies have calculated values for SSe for various surveillance system types in different countries; we used these values of SSe to construct distributions for each study's results, and then took random samples were taken from each relevant distribution and merged these random samples to produce one overall distribution for each surveillance type (passive, active or both for HPAI and LPAI). For example, the overall distribution for HPAI passive surveillance was generated from resampling from individual distributions for Spain, New Zealand, Nigeria and Denmark [33–35]. An exponential distribution (the best fit), was then fitted to the overall distributions for each surveillance type using the dfittool in MATLAB R2012b (The MathWorks Inc., Natick, MA, 2000) Distributions were generated for countries implement-

ing active surveillance for HPAI and LPAI, passive surveillance (HPAI only) or both surveillance types (see Figure 1 in main text).

Each country in the world was then assigned the relevant distribution according to the category of surveillance reported by each to the World Organisation for Animal Health (OIE) over the preceding three years, and via a project-specific surveillance survey [38] (see supplementary file Final Country Surveillance.csv; country designations and boundaries were set by using the Admin Boundaries shapefile supplied by the FAO gridded livestock database at `http://www.fao.org/ag/againfo/resources/en/glw/GLW_dens.html`). Information on AI was available from either or both sources for 169 of 242 countries. Of these 169 countries, only 26/91 (28.5%) countries presented the same information via both sources. Data conflicts involved 25 countries that had reported specific surveillance for LPAI to the OIE, but according to their survey results, were not undertaking any surveillance for LPAI. Additional information was provided by the OIE in the absence of survey results for either or both HPAI and LPAI in 24 cases, and was provided by the survey in the absence of OIE reports in 47 cases. In order to be able to update this information in the future, it was decided to classify countries according to their OIE status, except where missing information could be supplied from the survey results (i.e. when the two sources were directly contradicting, the OIE status was used). Countries reporting both types of surveillance were assigned to the active surveillance category (the most sensitive), and those with no known surveillance type were not included in the model. The classification of surveillance systems by country is shown in Figure 2.

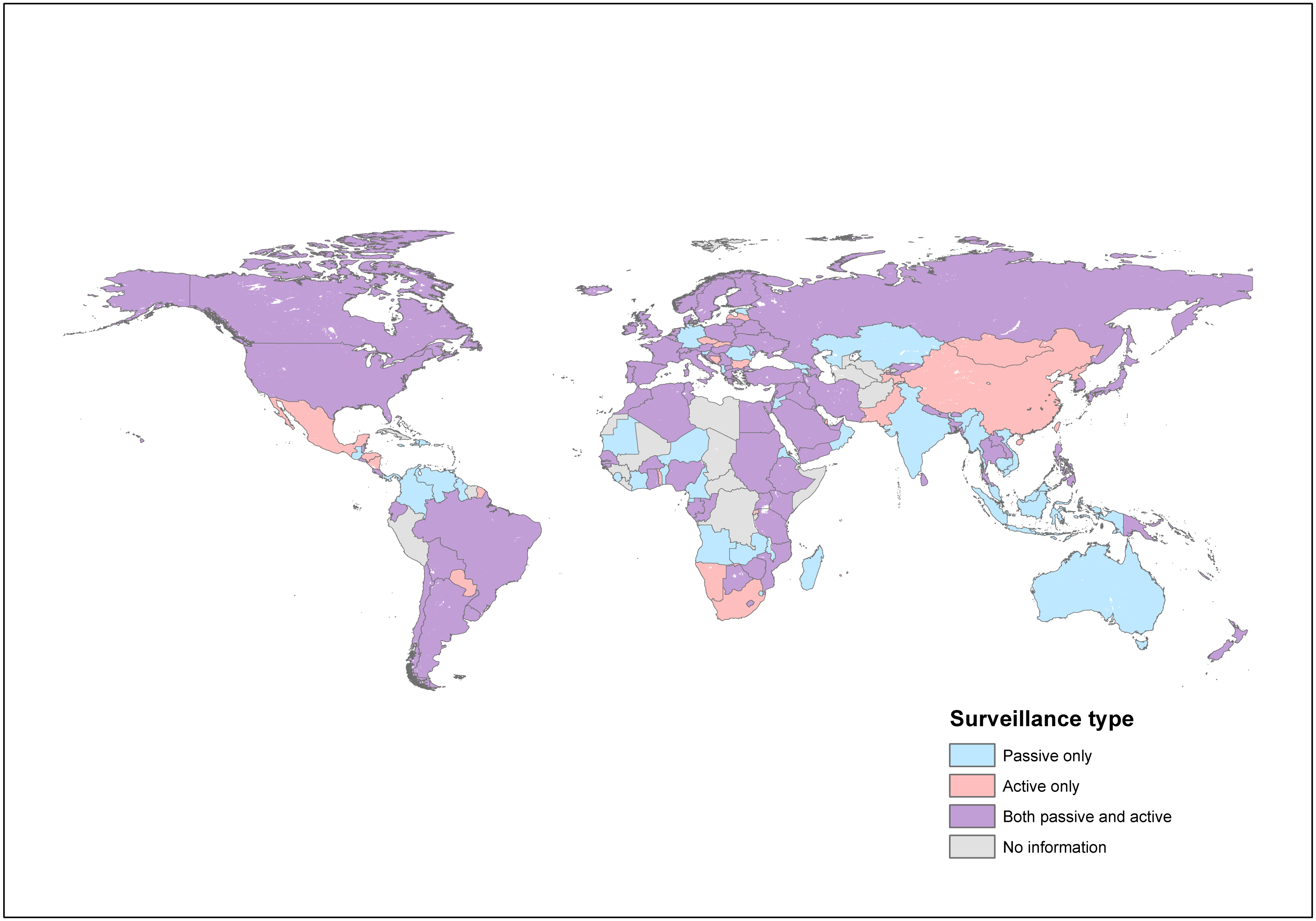

Figure 2. Classification of surveillance systems by country

Estimation of the relative fitness of a virus to cause human infection given effective exposure, $V(i)$

A full description of the calculation of the 32 virus score profiles is given in [17]. The virus scores are given in Table 3.

Table 3. Final virus scores for the 32 virus profiles. Virus profile relates to: receptor binding; presence of mutations in RNP; reassortment within virus; stalk deletions in NA; phylogenetic relatedness of HA.

Virus profile #	Receptor preference	Presence of known mutations in RNP complex	Re-assortment	Stalk deletion in NA	Phylogenetic relatedness of HA	Virus score, $V(i)$
1	$\alpha2,6$	None	None	Full stalk	Different	0.40
2	$\alpha2,6$	None	None	Full stalk	Related	0.45
3	$\alpha2,6$	None	None	Short stalk	Related	0.33
4	$\alpha2,6$	None	None	Short stalk	Different	0.38
5	$\alpha2,6$	None	Acquistion	Full stalk	Different	0.69
6	$\alpha2,6$	None	Acquistion	Full stalk	Related	0.74
7	$\alpha2,6$	None	Acquistion	Short stalk	Different	0.62
8	$\alpha2,6$	None	Acquistion	Short stalk	Different	0.67
9	$\alpha2,6$	Mutations	None	Full stalk	Different	0.65
10	$\alpha2,6$	Mutations	None	Full stalk	Related	0.71
11	$\alpha2,6$	Mutations	None	Short stalk	Related	0.59
12	$\alpha2,6$	Mutations	None	Short stalk	Different	0.64
13	$\alpha2,6$	Mutations	None	Full stalk	Different	0.94
14	$\alpha2,6$	Mutations	None	Full stalk	Related	1.00
15	$\alpha2,6$	Mutations	None	Short stalk	Different	0.88
16	$\alpha2,6$	Mutations	None	Short stalk	Related	0.93
17	$\alpha2,3$	None	None	Full stalk	Different	0.07
18	$\alpha2,3$	None	None	Full stalk	Related	0.12
19	$\alpha2,3$	None	None	Short stalk	Related	0.00
20	$\alpha2,3$	None	None	Short stalk	Different	0.06
21	$\alpha2,3$	None	Acquistion	Full stalk	Different	0.36
22	$\alpha2,3$	None	Acquistion	Full stalk	Related	0.41
23	$\alpha2,3$	None	Acquistion	Short stalk	Different	0.29
24	$\alpha2,3$	None	Acquistion	Short stalk	Different	0.35
25	$\alpha2,3$	Mutations	None	Full stalk	Different	0.32
26	$\alpha2,3$	Mutations	None	Full stalk	Related	0.38
27	$\alpha2,3$	Mutations	None	Short stalk	Different	0.26
28	$\alpha2,3$	Mutations	None	Short stalk	Related	0.31
29	$\alpha2,3$	Mutations	Acquistion	Full stalk	Different	0.62
30	$\alpha2,3$	Mutations	Acquistion	Full stalk	Related	0.67
31	$\alpha2,3$	Mutations	Acquistion	Short stalk	Different	0.55
32	$\alpha2,3$	Mutations	Acquistion	Short stalk	Related	0.60

Scores for case study viruses

As the epidemiological information for H5N1 isolates from the Empres-i database was not matched to online genetic sequence databanks (e.g. the GISAID online database), relevant GenBank accession numbers had to be matched by hand via inspection of the characteristics of each individual isolate. Hence, an initial subsample of 37 H5N1 Clade 1 and Clade 2 isolates with both clade and co-ordinate data were taken in order to identify the general characteristics of the five virus score factors for each clade (see Table 2 in main text). Thirty-three of the 37 isolates could be matched to GenBank accession numbers, although only 24 of the 33 strains held sufficient data within the GenBank database to identify all five virus score characteristics. In the final analysis, the virus score for H5N1 Clade 1 and Clade 2 was produced by analysing the characteristics of 17 and 2 isolates respectively. The characteristic of each virus factor was established by reading the published paper(s) linked with the isolate(s). Hence, no bespoke analysis of the genetic sequence was necessary for most isolates, which facilitated rapid scoring of viruses.

All isolates under each clade category were identically characterised, except for one isolate under H5N1 clade category 1 (which was judged not to have any significant genetic mutations in contrast to all other Clade 1 isolates). See Table 2 in main text.

A similar methodology was followed for the H7N9 isolates from the current 2013-2014 outbreak. Using the epidemiological information from an extract of all H7N9 isolates from the Empres-i database between 26th February 2013 and 4th April 2014, we matched 22 isolates to the GISAID sequence databases (see Table 4). The smaller number of H7N9 isolates meant it was much easier to match the correct GenBank accession number/s to each isolate within the Empres-i database. However, there were still many isolates that could not be matched due to insufficient data, or because no unique match existed. Despite much better links between databases for historic H5N1 isolates, only 31% (22/70) isolates were able to be confidently matched, of which 14 were isolated from birds or the environment (the bottom eight in Table 4 were human isolates).

Table 4. Profile of virus score characteristics for matched H7N9 strains

Strain	Collection date	Receptor preference	Presence of known mutations in RNP complex	Reassortment within virus	Stalk deletion in NA	Phylogentic relatedness of HA	Profile (see Table 3)	Normalised virus score (5th, 95th percentile)
A/chicken/Guangdong/SD641/2013	5/3/13	α2-6	None	Acquisition	Short stalk	Different	7	0.60 (0.58,0.61)
A/chicken/Jiangxi/SD001/2013	5/3/13	α2-6	None	Acquisition	Short stalk	Different	7	0.60 (0.58,0.61)
A/chicken/Zhejiang/SD007/2013	4/22/13	α2-3	None	Acquisition	Short stalk	Different	23	0.30 (0.23,0.36)
A/duck/Anhui/SC702/2013	4/16/13	α2-6	None	Acquisition	Short stalk	Different	7	0.60 (0.58,0.61)
A/duck/Zhejiang/SC410/2013	4/16/13	α2-6	None	Acquisition	Short stalk	Different	7	0.60 (0.58,0.61)
A/environment/Fujian/SC337/2013	4/30/13	α2-6	None	Acquisition	Short stalk	Different	7	0.60 (0.58,0.61)
A/environment/Henan/SC232/2013	4/24/13	α2-6	None	Acquisition	Short stalk	Different	7	0.60 (0.58,0.61)
A/environment/Henan/SD429/2013	4/24/13	α2-3	None	Acquisition	Short stalk	Different	23	0.30 (0.23,0.36)
A/environment/Shandong/SD038/2013	5/3/13	α2-6	None	Acquisition	Short stalk	Different	7	0.60 (0.58,0.61)
A/environment/Shandong/SD039/2013	5/3/13	α2-6	None	Acquisition	Short stalk	Different	7	0.60 (0.58,0.61)
A/environment/Shandong/SD049/2013	5/3/13	α2-6	None	Acquisition	Short stalk	Different	7	0.60 (0.58,0.61)
A/pigeon/Shanghai/S1069/2013	4/2/13	α2-6	None	Acquisition	Short stalk	Different	7	0.60 (0.58,0.61)
A/pigeon/Shanghai/S1421/2013	4/3/13	α2-6	None	Acquisition	Short stalk	Different	7	0.60 (0.58,0.61)
A/pigeon/Shanghai/S1423/2013	4/3/13	α2-6	None	Acquisition	Short stalk	Different	7	0.60 (0.58,0.61)
A/Zhejiang/2/2013	4/3/13	α2-6	Mutations	Acquisition	Short stalk	Different	15	0.84 (0.78,0.89)
A/Zhejiang/1/2013	3/24/13	α2-6	Mutations	Acquisition	Short stalk	Different	15	0.84 (0.78,0.89)
A/Hangzhou/3/2013	4/2/13	α2-6	Mutations	Acquisition	Short stalk	Different	15	0.84 (0.78,0.89)
A/Hangzhou/2/2013	3/25/13	α2-6	Mutations	Acquisition	Short stalk	Different	15	0.84 (0.78,0.89)
A/Hangzhou/1/2013	3/24/13	α2-6	Mutations	Acquisition	Short stalk	Different	15	0.84 (0.78,0.89)
A/Anhui/1/2013	3/20/13	α2-6	Mutations	Acquisition	Short stalk	Different	15	0.84 (0.78,0.89)
A/Shanghai/2/2013	3/5/13	α2-6	Mutations	Acquisition	Short stalk	Different	15	0.84 (0.78,0.89)
A/Shanghai/1/2013	2/26/13	α2-3	Mutations	Acquisition	Short stalk	Different	31	0.53 (0.43,0.64)

In contrast to the H5N1 isolates, the characteristics of the H7N9 isolates were quite varied, and four distinct profiles were identified. This result is not unexpected given that previous genotyping of H7N9 isolates have showed at least four different genotypes [12,13], but also because the small number of isolates are from disparate sources such as chickens, pigeons and the environment.

Model implementation

The model was written in the open-source software R (version 2.15.2) (`http://www.R-project.org`), using several GIS and logic model packages available: Raster, Maptools, RGDAL, BigMemory (and associated packages), KernSmooth and MLogit.
